# CCR5 interaction with HIV-1 Env contributes to Env-induced depletion of CD4 T cells in vitro and in vivo

**DOI:** 10.1186/s12977-016-0255-z

**Published:** 2016-03-29

**Authors:** Li-Chung Tsao, Haitao Guo, Jerry Jeffrey, James A. Hoxie, Lishan Su

**Affiliations:** Curriculum of Genetics and Molecular Biology, University of North Carolina at Chapel Hill, Chapel Hill, NC USA; Lineberger Comprehensive Cancer Center, University of North Carolina at Chapel Hill, Chapel Hill, NC USA; Department of Microbiology and Immunology, University of North Carolina at Chapel Hill, Chapel Hill, NC USA; Department of Medicine, University of Pennsylvania, Philadelphia, PA USA

**Keywords:** CCR5, HIV-1 pathogenesis, HIV-1 Env, Bystander CD4 T cells, Humanized mice

## Abstract

**Background:**

CD4 T cell depletion during HIV-1 infection is associated with AIDS disease progression, and the HIV-1 Env protein plays an important role in the process. Together with CXCR4, CCR5 is one of the two co-receptors that interact with Env during virus entry, but the role of CCR5 in Env-induced pathogenesis is not clearly defined. We have investigated CD4 T cell depletion mechanisms caused by the Env of a highly pathogenic CXCR4/CCR5 dual-tropic HIV-1 isolate R3A.

**Results:**

We report here that R3A infection induced depletion of both infected and uninfected “bystander” CD4 T cells, and treatment with CCR5 antagonist TAK-779 inhibited R3A-induced bystander CD4 T cell depletion without affecting virus replication. To further define the role of Env-CCR5 interaction, we utilized an Env-mutant of R3A, termed R3A-5/6AA, which has lost CCR5 binding capability. Importantly, R3A-5/6AA replicated to the same level as wild type R3A by using CXCR4 for viral infection. We found the loss of CCR5 interaction resulted in a significant reduction of bystander CD4 T cells death during R3A-5/6AA infection, whereas stimulation of CCR5 with MIP1-β increased bystander pathogenesis induced by R3A-5/6AA. We confirmed our findings using a humanized mouse model, where we observed similarly reduced pathogenicity of the mutant R3A-5/6AA in various lymphoid organs in vivo.

**Conclusion:**

We provide the first evidence that shows CCR5 interaction with a dual-tropic HIV-1 Env played a significant role in Env-induced depletion of CD4 T cells.

**Electronic supplementary material:**

The online version of this article (doi:10.1186/s12977-016-0255-z) contains supplementary material, which is available to authorized users.

## Background

The depletion of CD4 T cells is a hallmark of HIV-1 pathogenesis and AIDS progression [1]. Multiple studies have attempted to explain the depletion of CD4 T cells during HIV-1 infection. This can be broadly categorized into killing of uninfected “bystander” CD4 T cells, and “direct” killing of HIV-infected CD4 T cells [2]. Interestingly, the rate of CD4 T cell decline is discordant with low level of productively infected cells in HIV-positive individuals. This suggests “direct” death of productively infected cells contributes minimally towards AIDS progression, and bystander cell killing appears to be the leading cause for CD4 T cell loss and AIDS progression [3]. The HIV-1 Env, also known as the gp120/gp41 glycoprotein, is expressed on the surface of infected cells or on HIV-1 virions and can interact with bystander cells expressing CD4. This interaction is critical for HIV-1 entry and has been proposed to induce bystander CD4 T cell death [4–6].

HIV-1 strains can be broadly divided into two groups based on their *Env* tropism, each using CCR5 or CXCR4 chemokine co-receptor for viral entry. The CCR5-tropic HIV-1 Env interacts with CD4 and CCR5, infects CCR5+ CD4 T cells and macrophages, and is sensitive to CCR5 antagonists such as TAK-779. Likewise, the CXCR4-tropic virus interacts with CD4 and CXCR4, infects CXCR4+ CD4 T cells, and is sensitive to CXCR4 antagonists such as AMD-3100 [7, 8]. In addition, dual-tropic HIV-1 strains have been reported that are capable to utilize both CCR5 and CXCR4 for entry [9–12]. R5-tropic HIV-1 dominates during the early stages of HIV-1 infection. In later stages of infection, X4-tropic viruses emerge and are thought to be responsible for the accelerated decline of CD4 T cells and AIDS progression [13]. The highly pathogenic phenotype of late stage X4-viruses has been related to the abundant expression of CXCR4 in virtually all CD4 T cells, whereas CCR5-expressing CD4 T cells are mostly memory T cells [14]. However, in a significant proportion (>50 %) of AIDS patients, there is no co-receptor switch detected and their AIDS associated viruses are exclusively R5-tropic [15, 16]. Therefore, CCR5-tropic HIV-1 viruses can lead to AIDS progression but the mechanism remains unclear.

Previous reports have studied the pathogenic effect of HIV-1 Env binding to CCR5 by overexpression of R5-tropic Env on cell surface or by using recombinant R5-tropic gp120 proteins [4, 5, 17]. However, the pathogenic effect of R5-tropic Env has not been studied in HIV-1 infection models, or directly compared to HIV-1 viral load. In this report, we studied the Env pathogenicity of a highly pathogenic dual-tropic HIV-1 strain (R3A) derived from a rapid progressor [9]. The *Env* gene of R3A is highly pathogenic and has been used for HIV-1 pathogenesis studies [9–11]. The interaction of the V3 region of R3A-Env with the co-receptors and its specificity for either CCR5 or CXCR4 has been elucidated in a previous study [8]. We took advantage of a mutant R3A strain termed R3A-5/6AA from the study, which has lost the ability to bind and utilize CCR5 but can still use CXCR4 for viral infection, therefore not affecting viral replication capability. Interestingly, the mutant R3A-5/6AA is substantially less pathogenic then the wild type R3A, as evidenced by the reduction of virus-mediated bystander CD4 T cells depletion. Supporting the functional relevance of CCR5 interaction by R3A-Env in CD4 T cells pathogenesis, we found that the inhibition of Env-CCR5 binding by CCR5 antagonistic drug TAK-779 reduced R3A-induced bystander CD4 T cells killing, whereas stimulation of the CCR5 receptor with agonistic drug MIP-1β increased the pathogenesis effect. We confirmed our findings in vivo using a humanized mouse model, and we observed reduced bystander pathogenesis of the mutant R3A-5/6AA compared to the wild type R3A infection in CD4 T cells in the blood, spleen and bone marrow. We provide the first evidence in two physiologically relevant HIV-1 infection models that shows CCR5 interaction with a dual-tropic HIV-1 Env plays a significant role in Env-induced depletion of bystander CD4 T cells.

## Results

A highly pathogenic HIV-1 isolate R3A induces depletion of both productively infected cells and bystander CD4 T cells in activated PBMCs.

**Fig. 1:**
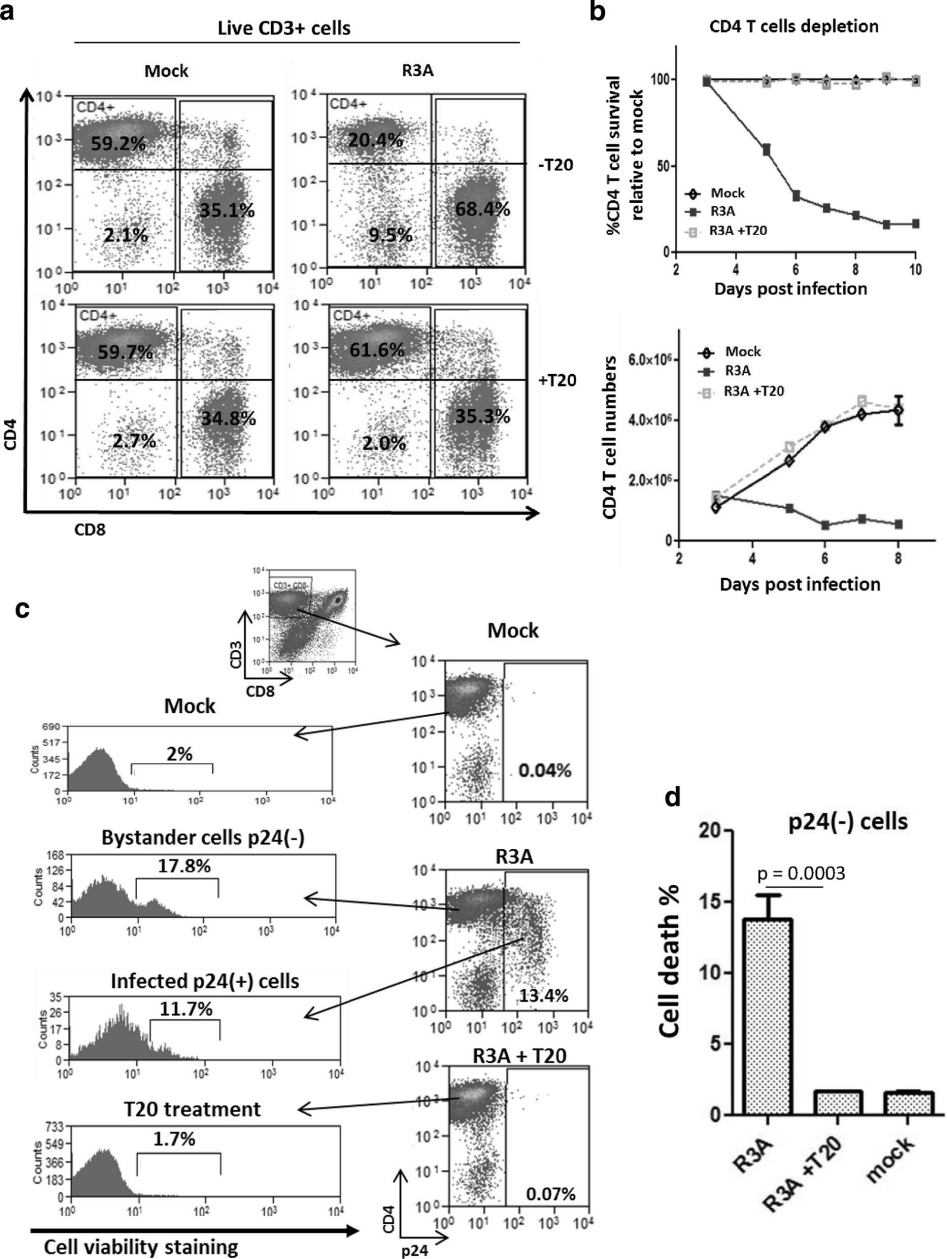
A highly pathogenic HIV-1 isolate R3A induces depletion of both productively infected and bystander CD4 T cells. **a** HIV-R3A infection in PBMC leads to efficient depletion of CD4+T cells. PBMCs were infected with R3A virus, and stimulated with anti-CD3/CD28/CD2 activation beads at 3 h post infection and cultured in the presence of IL-2 (20 U/mL). FACS Plots show CD4 and CD8 staining of populations gated on live CD3(+) T cells in PBMC at 6 days post infection (dpi). HIV-1 fusion inhibitor T20 was used as negative control to prevent HIV-1 infection. **b** Graphical summary showing kinetics of CD4 T cells depletion by R3A from 3 to 10 dpi. CD4 T cells are identified as live CD8 (−) CD3(+) population. Data are presented as %CD4 T cell survival relative to mock infection (*top*) and total CD4 T cell numbers (*bottom*) over time. **c** FACS gating strategies are presented. CD4 T cells were identified as CD3(+) CD8(−) population. Uninfected bystander CD4 T cells were identified as p24(−) cells and infected CD4 T cells as p24(+) cells (*right graphs*). Cell viability of both bystander and infected populations was quantified by co-staining with a cell viability dye (*left graphs*). **d** Graphical summary of bystander p24(−) cell death at 6 days post infection by R3A. *p < 0.05

We used a primary activated PBMC culture infection model to study the pathogenesis of the highly pathogenic dual-tropic HIV-1 strain (termed R3A) on CD4 T cells. Briefly, freshly isolated PBMCs (peripheral blood mononuclear cells) were infected with an MOI of 0.01 for 3 h, followed by T cell stimulation with CD3/CD28 activation beads. The kinetics of virus-induced CD4 T cell depletion can be accurately monitored (Fig. 1a), as CD4 T cell percentages and numbers in R3A infected PBMCs gradually decreased over time (Fig. 1b). R3A pathogenesis was completely prevented by the fusion inhibitor T20, which effectively inhibited viral entry and replication as measured by intracellular HIV-1 p24 staining (Fig. 1c). Co-staining of intracellular p24 with a cell viability dye allowed us to analyze the percentage of dying cells in both productively infected and uninfected CD4 T cells (Fig. 1c). Compared to mock infection, we observed a significant higher percentage of dying cells in the p24(−) CD4 T cell populations at 6 days post R3A infection, therefore we termed it as bystander cell pathogenesis (Fig. 1d).

### CCR5 antagonist TAK-779 protects CD4 T cells from R3A-induced bystander CD4 T cell depletion

**Fig. 2:**
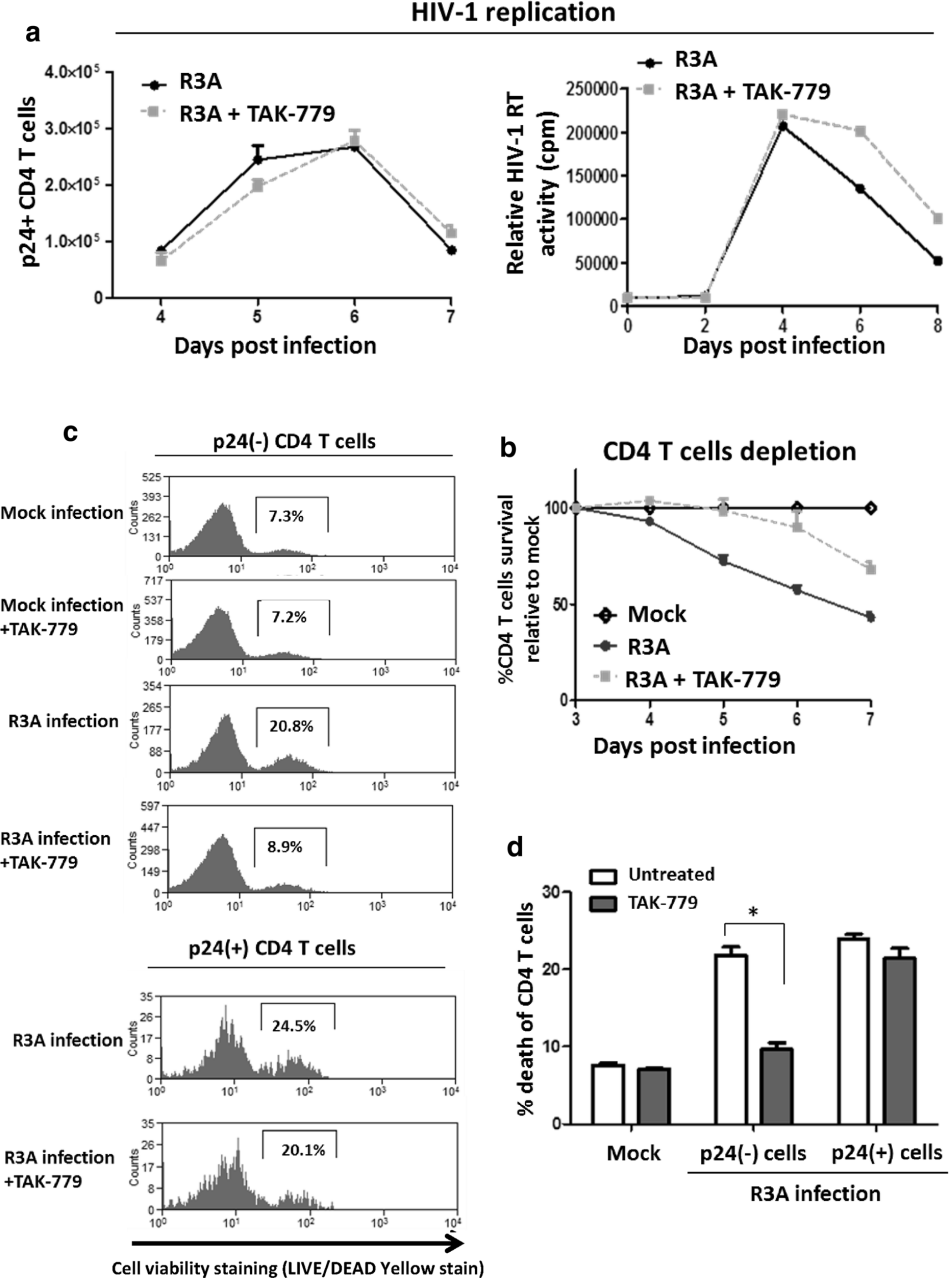
CCR5 antagonist TAK-779 protects CD4 T cell from R3A-induced bystander CD4 T cell depletion a PBMCs were treated with CCR5 antagonist TAK-779 (5 μM) before infection with R3A and maintained after infection. HIV-1 viral replication in CD4 T cells was measured by intracellular p24 staining or by extracellular HIV-1 reverse transcriptase levels. **b** CD4 T cell depletion by R3A in the presence of TAK-779 was measured by FACS analysis as described in Fig. 1b. %CD4 T cells over time relative to mock infected PBCMCs are presented. **c** Cell viability of bystander and infected CD4 T cells during R3A infection in the presence of TAK-779 was analyzed as described in Fig. 1c. **d** Graphical summary of viral induced bystander cell death from **c**, presented as cell death percentage of p24(−) CD4 T cells at 6 days post infection. Experimental results were repeated with PBMCs from 2 different blood donors. *p < 0.05

Expression of R5-tropic HIV-1 Env proteins on cell surface could induce the depletion of neighboring SupT1 cells in a CCR5-dependent manner [5]. To investigate how CCR5 interaction during dual-tropic R3A infection contributes to CD4 T cell depletion, we treated R3A infected cells with the CCR5 antagonist TAK-779. TAK-779 is known to block HIV-1 Env interaction with CCR5 and inhibit the infections of CCR5-tropic viruses [18]. As the dual-tropic R3A can also utilize CXCR4 for infection, we found R3A replication in CD4 T cells wasn’t significantly affected by TAK-779 treatment, as measured by intracellular p24 staining or extracellular HIV-1 RT levels (Fig. 2a). Interestingly, CD4 T cell depletion was significantly reduced by TAK-779 treatment (Fig. 2b). Furthermore, TAK-779 significantly reduced (p24−) CD4 T cell death to near background levels (Fig. 2c, d). However, cell death of HIV-1 infected (p24+) CD4 T cells was not significantly affected by TAK-779 (Fig. 2c, d). This data indicates blocking R3A-Env interaction with CCR5 reduced the pathogenesis on bystander CD4 T cells.

The binding of HIV-1 Env to the other co-receptor, CXCR4, has also been implicated in Env-induced cell death [19]. We therefore tested how CXCR4 binding by R3A Env affects viral pathogenesis using AMD3100, a CXCR4 antagonist [20]. Unlike TAK-779, the blockage of CXCR4 usage by AMD3100 significantly reduced the entry and replication of R3A in PBMCs (Additional file 1: Fig S1a, b). As expected, the combined treatment of TAK-779 and AMD3100 completely inhibited R3A infection, similar to T20. This suggests the dual-tropic R3A, although capable of using either co-receptor for entry, relies more heavily on CXCR4 for replication in primary CD4 T cells. Although blocking CXCR4 usage by R3A also reduced CD4 T cell depletion (Additional file 1: Fig S1c), the interpretation here is difficult since virus replication was also significantly inhibited.

### Ablation of CCR5 usage reduces dual-tropic HIV-1 pathogenesis in PBMC infection

**Fig. 3:**
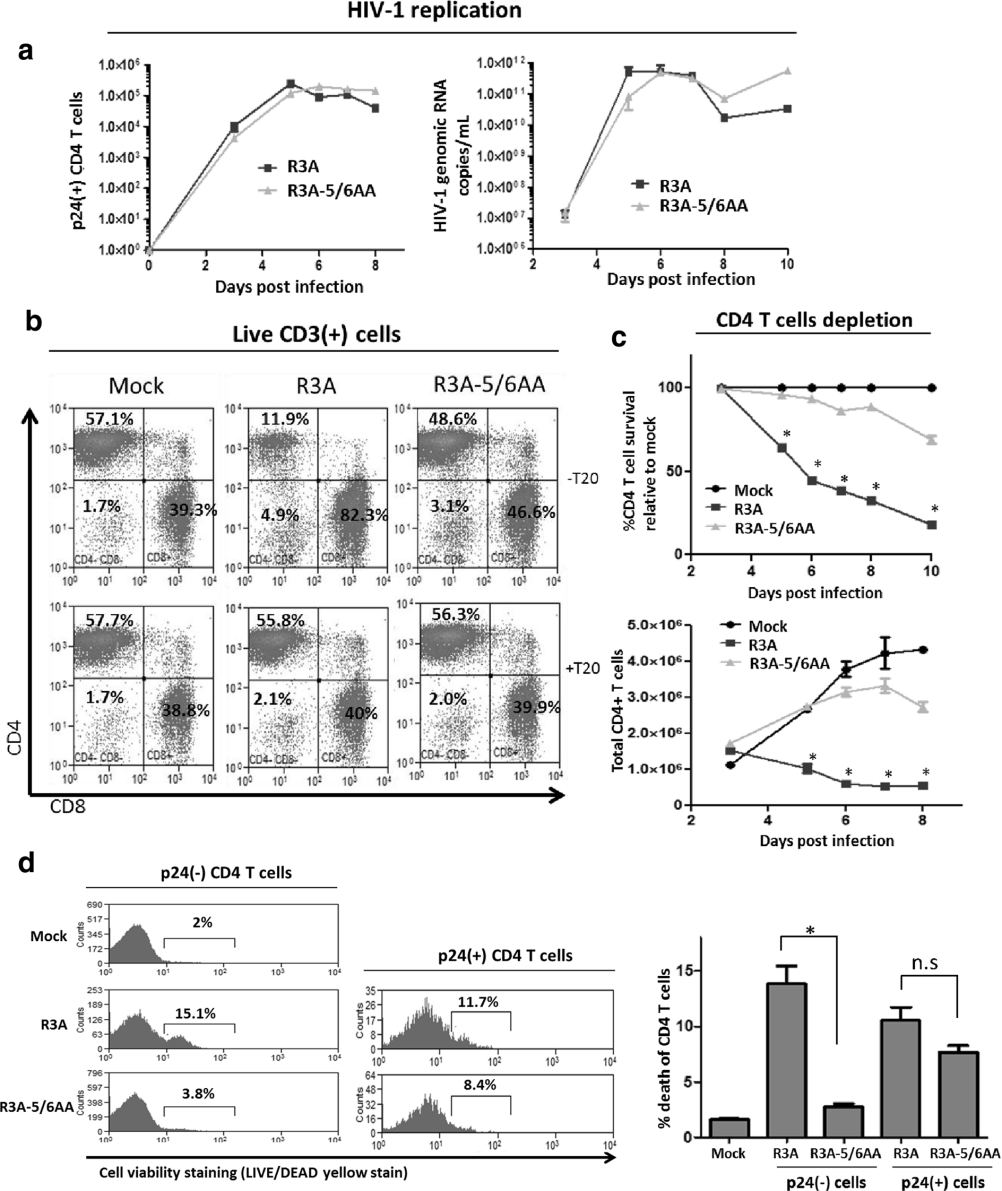
Ablation of CCR5 usage reduces dual-tropic HIV-1 pathogenesis in PBMC infection. **a** Intracellular p24 staining and FACS analysis was used to quantify viral replication in infected PBMCs. Infected (p24+) CD4 T cell numbers were calculated by multiplying the percentage of (p24+) cells with total CD4 T cell numbers at each time point (*left*). RT-qPCR analysis of HIV-1 genomic RNA levels was used to measure extracellular viral replication of R3A and R3A-5/6AA in infected PBMCs (*right*). **b** Representative FACS Plots are presented showing CD4 and CD8 populations gated on live CD3(+) T cells in PBMC at 6 days post infection. HIV-1 fusion inhibitor T20 treatment was used as negative control. **c** Kinetics of CD4 T cell depletion by R3A versus R3A-5/6AA was analyzed as described in Fig. 1b. CD4 T cell survival are measured as %CD4 T cells in infected PBMCs relative to mock infection (*top*), or live CD4 T cell numbers (*bottom*) over time. **d** p24(−) and p24(+) CD4 T cell death in R3A and R3A-5/6AA infected PBMCs are quantified at 6 days post infection, as described in Fig. 1C. FACS plots (*left*) and graphical summary (*Right*) from a representative experiment are presented. Experimental results were repeated with PBMCs from 3 different blood donors. *p < 0.05

Our results with CCR5 antagonist TAK-779 treatment suggest the interaction of R3A-Env with CCR5 receptor contributed to CD4 T cells depletion during R3A infection. To expand this finding, we used a genetic mutant of R3A that is incapable of binding to the CCR5 receptor. As published before [8], the R3A-5/6AA mutant (with two alanine substitutions in the 5th and 6th amino acids of Env V3 region) has lost its capability to infect CCR5-expressing cells but not CXCR4-expressing cells (Additional file 2: Fig. S2a). R3A-5/6AA could efficiently utilize CXCR4 for virus entry and replication. Accordingly, we observed the virus replication was not significantly affected by its ablation of CCR5 usage, as R3A-5/6AA replicated to wild type R3A viremia levels as measured by both intracellular p24 staining and extracellular HIV-1 genomic RNA levels (Fig. 3a; Additional file 2: Fig. S2b). We then compared the pathogenesis of wild-type R3A with R3A-5/6AA. As shown before, the highly pathogenic R3A can significantly deplete CD4 T cell levels, with 60 % depletion at 6 dpi and 90 % depletion at 10 dpi. Remarkably, ablation of CCR5 usage by the mutant R3A-5/6AA significantly reduced CD4 T cell pathogenesis, with only 10 % depletion at 6 dpi and 35 % depletion at 10 dpi (Fig. 3b, c). This indicates R3A-Env binding with CCR5 strongly contributed to Env-mediated CD4 T cell depletion. The reduced pathogenicity of R3A-5/6AA observed is not a consequence of reduced virus growth advantage, as R3A-5/6AA replicated to similar levels as R3A (Fig. 3a). As shown before, R3A infection reduced the viabilities of both p24(−) and p24(+) CD4 T cells (Fig. 1c). Interestingly, the ablation of CCR5 binding in R3A-5/6AA infection completely rescued death of p24(−) bystander CD4 T cells to background levels (Fig. 3d). The cell viability of p24(+) cells was not significantly different in R3A and R3A-5/6AA infection (Fig. 3d). Therefore, our data indicate R3A could efficiently deplete both bystander and infected CD4 T cells, whereas R3A-5/6AA only induced perceivable pathogenesis in productively infected CD4 T cells. The reduced pathogenicity of R3A-5/6AA infection seen in Fig. 3c was therefore due to reduced bystander cell death, a consequence of the loss of CCR5 binding capability by the Env mutant. We conclude R3A-induced bystander pathogenesis is dependent on the viral Env interaction with CCR5.

### CCR5 agonist MIP-1β enhances R3A-5/6AA pathogenesis to promote bystander CD4 T cell depletion

**Fig. 4:**
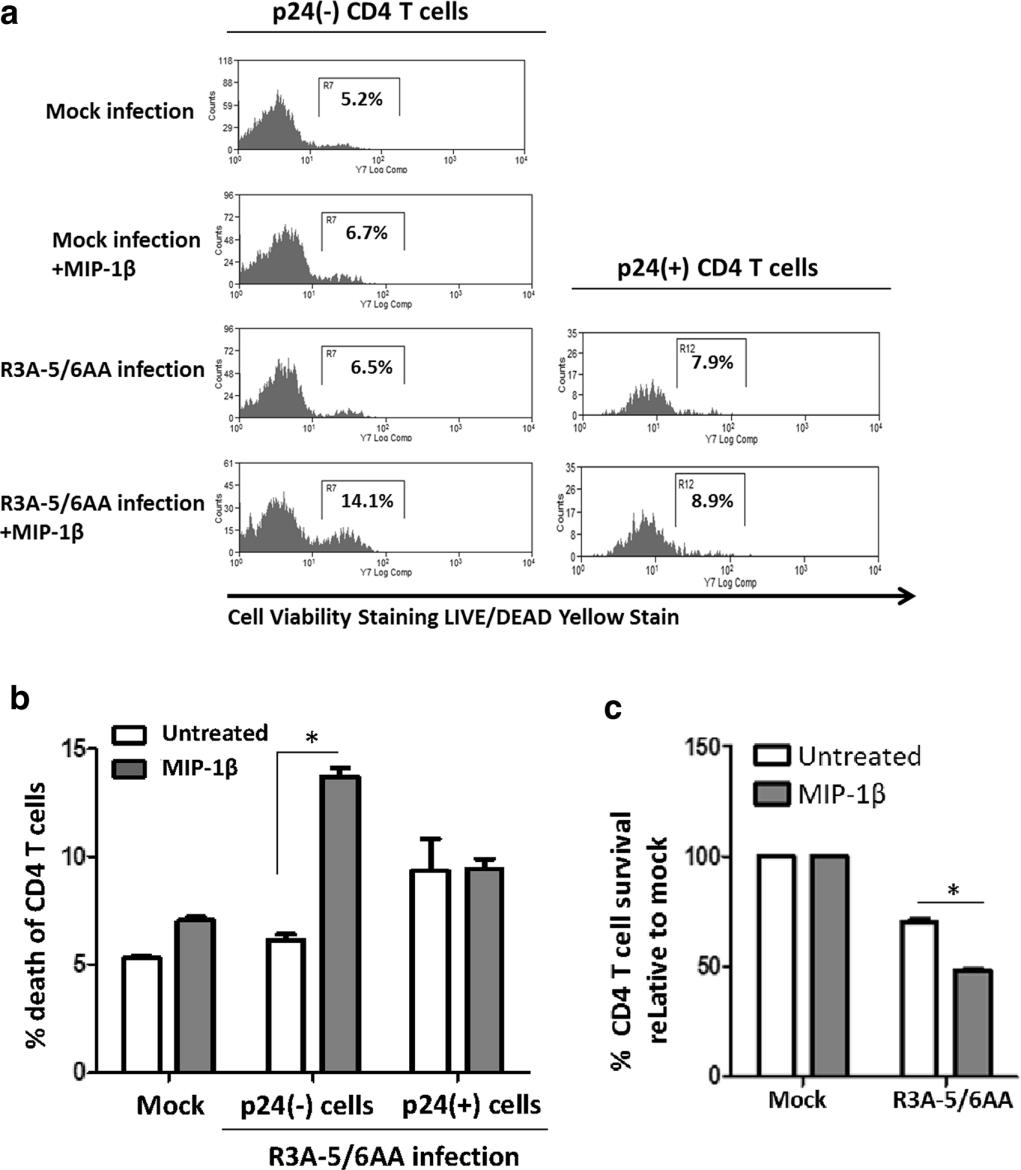
CCR5 agonist MIP-1β enhances R3A-5/6AA pathogenesis to promote bystander CD4 T cell depletion PBMCs were infected with R3A-5/6AA virus and treated with recombinant MIP-1 β (200 ng/mL) at 3 h post infection. At 6 days post infection, viabilities of p24(−) and p24(+) CD4 T cell were measured as described in Fig. 1c. **a** Representative FACS plots and **b** graphical summary are presented. **c** %CD4 T cells survival at 6 days post infection was measured by gating on live CD8(−) CD3(+) cells. *p < 0.05

MIP-1β is a CCR5-specific chemokine and agonist [21]. We investigated whether activation of CCR5 by MIP-1β could complement for the ablated Env-CCR5 interaction in R3A-5/6AA infection and therefore enhance bystander pathogenesis. We found MIP-1β treatment significantly increased the death of p24(−) CD4 T cells in R3A-5/6AA infected PBMC cultures, without affecting the viability of p24(+) CD4 T cells (Fig. 4a, b). Accordingly, MIP-1β also decreased the CD4 T cell survival (Fig. 4c). We conclude MIP-1β binding to CCR5 could partly mimic R3A’s Env interaction with CCR5 and increase bystander CD4 T cell depletion.

### Ablation of CCR5 usage reduces pathogenesis of dual tropic HIV-1 in a humanized mouse model in vivo

**Fig. 5:**
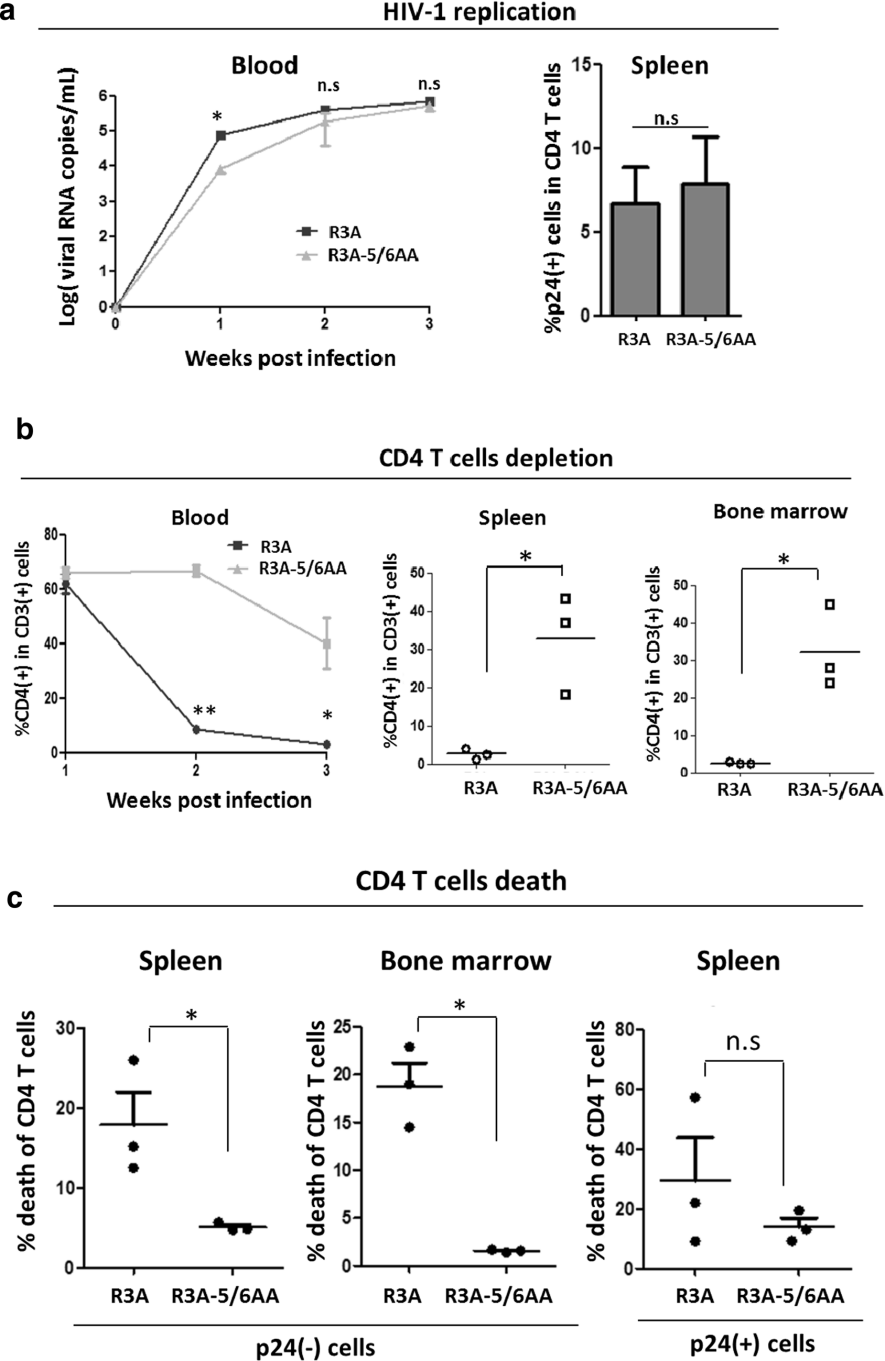
Ablation of CCR5 usage reduces pathogenesis of dual tropic HIV-1 in a humanized mouse model. **a** Humanized mice were infected with R3A or R3A-5/6AA as described in “Methods” section. Viral replication was assessed weekly by RT-qPCR measurement of HIV-1 genomic RNA in the blood. HIV-1 infection in CD4 T cells was analyzed by intracellular p24 staining of splenocytes isolated from infected mice at 3 weeks post infection (wpi). **b** Total PBMCs in blood, spleen and bone marrow from infected animals were harvested at 3wpi, and CD4 T cell depletion was analyzed by FACS staining as described in Fig. 1b. **c** p24(−) and p24(+) CD4 T cell viability in R3A versus R3A-5/6AA infection in the infected animal’s spleen and bone marrow were analyzed as described in Fig. 1c. *p < 0.05

To confirm that Env-CCR5 interaction contributes to HIV-1 bystander pathogenesis in a relevant HIV-1 infection model in vivo, we compared the infection and pathogenesis of R3A with R3A-5/6AA in the huHSC mice, a suitable small animal model to study HIV pathogenesis in vivo [22]. Similar to infected PBMC cultures, R3A-5/6AA replication was delayed initially at 1 week post infection but reached similar viremia levels as R3A at 2 and 3 weeks post infection as measured by HIV genomic RNA levels in the blood and intracellular p24 staining of CD4 T cells in the spleen (Fig. 5a). When analyzed at 3 weeks post infection, we found R3A-5/6AA was significantly less pathogenic than R3A in vivo, with reduced CD4 T cell depletion in the blood, spleen and bone marrow (Fig. 5b). Furthermore, R3A infection resulted in higher cell death of p24(-) CD4 T cells than R3A-5/6AA infection in vivo (Fig. 5c). The viability of infected p24(+) CD4 T cells was similar in R3A and R3A-5/6AA infection (Fig. 5c). These findings indicate R3A Env interaction with CCR5 induced bystander CD4 T cell pathogenesis in HIV-infected humanized mice.

## Discussion

Multiple viral and host factors determine the variability in HIV-1 disease progression [17, 23–26]. Cellular tropism and receptor/co-receptor usage for viral entry are major factors influencing HIV pathogenesis. Despite extensive research, the exact mechanism of how those factors contribute to the gradual loss of CD4 T cells is still enigmatic. Bystander CD4 T cell death plays a major contribution towards AIDS progression. A recent report has revealed a mechanism for HIV-induced CD4 T cell depletion, which involves abortive non-productive HIV infection in resting CD4 T cells, followed by IFI16 activation and caspase-1 dependent pyroptosis [27–29]. Besides the abortive RT products in non-productively infected resting cells, several HIV-1 proteins have also been reported to contribute to the depletion of bystander (uninfected and non-productively infected) CD4 T cells, including the Env [4, 5, 17, 19], Vpr [30], Nef [31, 32] and Tat [33]. The Env protein is of specific interest in mediating AIDS progression, and has been implicated as an important cytopathic determinant of AIDS-associated CCR5-tropic viruses [34, 35]. CCR5 expression levels on cell surface correlate with increased host susceptibility to R5-tropic Env-induced apoptosis [5]. Interestingly, humanized mice engrafted with CCR5Δ32^+/−^ donor cells supported HIV-1 replication with reduced CD4 T cell depletion [36]. It has been difficult to directly define the role of CCR5 interaction in HIV-induced CD4 T cell depletion because CCR5 interaction is required for the replication of CCR5-tropic HIV-1. Our findings using a dual-tropic HIV-1 confirmed that the interaction between CCR5 and HIV-1 Env is a critical determinant of bystander CD4 T depletion. The dual-tropic R3A virus is known to be highly pathogenic, depleting thymocytes through multiple mechanisms including fusion-induced apoptosis [10] and fusion-independent interferon-mediated mechanism [11]. In humanized mice, R3A infection caused rapid depletion of both infected and uninfected CD4 T cells [22, 37]. Using an *Env* mutant R3A-5/6AA that has lost interaction with CCR5, we were able to show that the ablation of CCR5 usage by the dual-tropic R3A virus effectively decreased viral pathogenicity on bystander CD4 T cells in activated PBMC cultures (Fig. 3) and in humanized mice (Fig. 5). Notably, ablation of CCR5 usage did not significantly affect the killing of productively infected CD4 T cells (Figs. 3d, 5c), corresponding with previous reports that the R3A Env fusion activity [10] and other HIV-1 proteins can promote death of directly infected cells [38, 39]. It is of note that the replication of mutant R3A-5/6AA was lower compared to R3A at early time points (Fig. 5a). The delayed replication peak may have contributed to the decreased pathogenicity of the mutant virus at early time points.

Signal transduction through Env-CCR5 interaction on bystander CD4 T cells may contribute to viral pathogenesis [17, 24]. Here we showed that CCR5 antagonist TAK-779 decreased R3A-induced bystander CD4 T cells depletion (Fig. 2), whereas CCR5 agonist MIP-1β increased bystander pathogenesis (Fig. 4), supporting a role for CCR5 stimulation/signaling in R3A pathogenesis. Mechanistically, HIV-1 Env binding to CCR5 induces a signaling cascade including calcium influx, Pyk2 phosphorylation and downstream activation of p38 MAPK pathway [40–42]. Using a p38 inhibitor, Li et al. has shown that CCR5 engagement by HIV-1 Env leads to p38 activation and Fas- and caspase-dependent cell death [43]. The signaling through CCR5 by the R3A Env and subsequent p38 activation, together with Env binding to CD4, may be involved in R3A induced bystander T cell killing. MIP-1β binds to CCR5 and is also known to similarly activate Pyk2 and p38 MAPK pathways [42, 44], which may mimic the Env-CCR5 interaction and contribute to bystander cell death, as shown in Fig. 4. In addition to CCR5, the interaction of CXCR4 with HIV-1 Env is also known to contribute to Env-mediated cell death [19]. Although treatment with CXCR4 antagonist AMD3100 decreased R3A pathogenesis, the interpretation in this study is difficult since AMD3100 also significantly inhibited R3A replication (Fig S1). Therefore, like most dual-tropic viruses, R3A relies more heavily on CXCR4 than CCR5 usage for infection in primary PBMC. It remains to be determined whether CXCR4 binding by R3A Env also contributes to CD4 T cell death during infection. Lastly, the cooperative engagement of R3A Env to both CCR5 and CXCR4 may result in stronger binding of the Env to CD4 T cells, which may directly affect Env-mediated cell death. Loss of CCR5 engagement in our studies may have resulted in a weaker binding to CD4/CXCR4 and therefore a reduced Env pathogenicity. Accordingly, MIP-1β may have enhanced this Env-CXCR4 interaction and therefore contributing to the increased cell death.

In summary, we provide the first evidence in relevant infection models with a dual tropic HIV-1 that can efficiently infect primary human PBMC when its binding to CCR5 is blocked genetically or pharmacologically, demonstrating CCR5 interaction with dual-tropic HIV-1 Env played a significant role in Env-induced depletion of bystander CD4 T cells. Our findings suggest that drugs such as Maraviroc or gene therapy targeting CCR5 interaction with HIV gp120 can not only prevent R5-mediated HIV-1 entry, they can also reduce Env-mediated CD4 T cell depletion and AIDS disease progression.

## Conclusion

We provide the first evidence in relevant infection models that shows CCR5 interaction with a dual-tropic HIV-1 Env played a significant role in Env-induced depletion of CD4 T cells.

## Methods

### Cell cultures

Total PBMCs were isolated from peripheral blood of healthy donors by Ficoll-Paque™ Plus (GE Healthcare) density gradient centrifugation and maintained in RPMI 1640 (Gibco) supplemented with 10 % FBS, 1X Antibiotic–Antimycotic (Invitrogen) and 1 µM l-glutamine. T cell activation in PBMCs were performed with CD3/CD2/CD28 activation beads (Miltenyi Biotech) and cultured in RPMI medium described above containing 20 U/mL recombinant human interleukin-2 (IL-2). HEK293T cells were cultured in DMEM (Gibco) containing 10 % FBS and 1X Antibiotic–Antimycotic. MAGI cells (NIH AIDS Research and Reference Reagent Program) were maintained in the same medium plus selection antibiotics [45].

### HIV-1 virus production

X4/R5 dual tropic HIV-1 (strain pNL4-R3A) was generated by cloning the R3A *Env* gene in the background of pNL4-3 proviral genome as previously described [9]. The mutant R3A proviral strain (pNL4-R3A-5/6AA) was generated and kindly provided by Dr. James Hoxie [8]. HIV-1 virions were produced by CaCl2-BES (*N,N*-bis[2-hydroxyethyl]-2-minoethanesulfonic acid) transfection of proviral plasmids in 293T cells. 293T cells cultured on a 10 cm plate were transfected with 30 μg DNA plasmids of pNL4-R3A or pNL4-R3A-5/6AA, and cell supernatants were harvested at 48 h post transfection and passed through 45 µm membrane filter. Concentration of virus stocks were measured by p24 ELISA assay (Frederick National Laboratory for Cancer Research—AIDS and Cancer Virus Program). Infectious titers were determined in CCR5-expressing or CXCR4-expressing MAGI cells as previously described [45].

### HIV-1 infection of PBMC cultures

Unless indicated otherwise, infections of PBMCs with R3A or R3A-5/6AA was performed by infecting 1 × 10^6^ freshly isolated PBMCs with MOI of 0.01 (as titered in CXCR4-expressing MAGI cells) for 3 h at 37 °C in a total volume of 100 µL supplemented RPMI medium containing polybrene (8 µg/mL). At 3 h post infection, viral inoculum were washed with PBS, activated with CD3/CD28 activation beads as described above, and cultured at concentration of 0.5 × 10^6^ cells/mL in the presence of IL-2 (20 U/mL). When indicated, PBMCs were treated with 10 µg/mL fusion inhibitor T20 (NIH AIDS Reagents Program) before infection and maintained throughout the experiment. TAK-779 and AMD3100 (NIH AIDS Reagents Program) were treated before infection at an IC90 dose of 5 and 2 µM respectively, as determined before in U373-CD4-CCR5/CXCR4 cells. Treatment with 200 ng/mL MIP-1β started at 3 h post infection and maintained.

### Flow cytometry analysis

Aliquots of infected PBMC samples (~1E5 cells) were collected for analysis at the indicated time points post HIV-1 infection. Staining for cellular surface CD3, CD4, CD8 (BD Biosciences) and viability dye (LIVE/DEAD fixable yellow dead stain, Invitrogen) were performed in 2 % FBS. Intracellular staining was performed after surface antibody staining, with the use of Fixation/Permeabilization Solution Kit (BD Cytofix/Cytoperm™), followed by staining with antibodies targeting p24 (Beckman Coulter, #KC-57). Cells were fixed by 1 % paraformaldehyde and analyzed with Cyan ADP FACS machine (DAKO). Live total cell numbers were counted by Guava EasyCytes after staining with viability dye. %CD4 T cells survived at a the time point were analyzed as live CD3(+) CD8(−) populations in PBMCs. Total live CD4 T cell numbers at a given time where calculated using the total live cell numbers multiplied by %CD4 T cells in PBMCs. Total infected CD4 T cell numbers were calculated using total CD4 T cell number multiplied by %p24(+) in live CD3(+) CD8(−) cells.

### HIV-1 infection of humanized mice and analysis

DKO-huHSC mice were constructed as previously reported [22]. DKO-huHSC mice were infected with HIV-1 at 4000 infectious units/mouse by intravenous injection. At termination, whole animal’s blood and lymphoid organs including spleen and bone marrow were harvested as described before [22]. Total lymphocytes were isolated from lymphoid organs and red blood cells were lysed with ACK buffer, and remaining cells were stained and fixed before flow cytometry analysis as described above.

### RT-qPCR analysis and HIV-1 reverse transcriptase activity analysis of viral load

Viremia in supernatants from infected PBMC cultures or from blood plasma of infected humanized mice was analyzed by viral RNA extraction (QIAamp viral RNA mini kit, Qiagen), and analyzed by RT-qPCR (Taqman One-Step RT-qPCR Master Mix, ABI) using the following primers and probe targeting HIV-1 Gag region: 765gagF 5′-GGTGCGAGAGCGTCAGTATTAAG-3′; 911gagR 5′-AGCTCCCTGCTTGCCCATA-3′; probe FAM-AAAATTCGGTTAAGGCCAGGGGGAAAGAA-QSY7 (TAMRA). HIV-1 RT activity was analyzed in the infected PBMC supernatant as described before in Lee MH et al., *J Clinical Microbiology,* 1987.

### Statistical analysis

The significance of all comparisons was calculated by the use of a Student 2-tailed *t* test, and results were considered significant when *p* < 0.05.

## Additional files

10.1186/s12977-016-0255-z CXCR4 antagonist AMD3100 inhibits R3A replication.a PBMCs were treated with the indicated drugs before infection with R3A, and viral infection efficiencies were measured by %p24+ cells with FACS analysis at 4 days post infection. AMD3100 (2µM) and TAK-779 (5µM) were used at >IC90 dose as determined before in U373-CD4-CCR5/CXCR4 cells. b PBMCs were treated with AMD3100 before infection with R3A and maintained after infection. HIV-1 replication was measured by extracellular HIV-1 reverse transcriptase activity in the cell supernatant. c CD4 T cell depletion by R3A in the presence of AMD3100 was measured by FACS analysis. %CD4 T cells relative to mock infected PBMCs are presented.
10.1186/s12977-016-0255-z Similar infection levels by R3A and R3A-5/6AA in PBMCs.a Infectivity of R3A and R3A-5/6AA HIV-1 strain was measured in CCR5-expressing (Top) or CXCR4-expressing (Bottom) U373 MAGI cells. Viral infectivity was presented as infectious units per ng of p24. b PBMCs were infected with 20ng p24 of R3A or R3A-5/6AA virus stock, and viral replication kinetics was analyzed by intracellular p24 staining. Representative FACS plots of p24 staining at 6dpi are presented. Supplementary to Fig. 3a.
