# Supplementary material for: CCR5 interaction with HIV-1 Env contributes to Env-induced depletion of CD4 T cells in vitro and in vivo
Source: Retrovirology. 2016 Mar 29;13:22. doi: 10.1186/s12977-016-0255-z (PMC4812640; doi:10.1186/s12977-016-0255-z)
Supplement: Supplementary file 1 — 10.1186/s12977-016-0255-z CXCR4 antagonist AMD3100 inhibits R3A replication.a PBMCs were treated with the indicated drugs before infection with R3A, and viral infection efficiencies were measured by %p24+ cells with FACS analysis at 4 days post infection. AMD3100 (2µM) and TAK-779 (5µM) were used at >IC90 dose as determined before in U373-CD4-CCR5/CXCR4 cells. b PBMCs were treated with AMD3100 before infection with R3A and maintained after infection. HIV-1 replication was measured by extracellular HIV-1 reverse transcriptase activity in the cell supernatant. c CD4 T cell depletion by R3A in the presence of AMD3100 was measured by FACS analysis. %CD4 T cells relative to mock infected PBMCs are presented. [file 12977_2016_255_MOESM1_ESM.pdf]

**Figure S1**

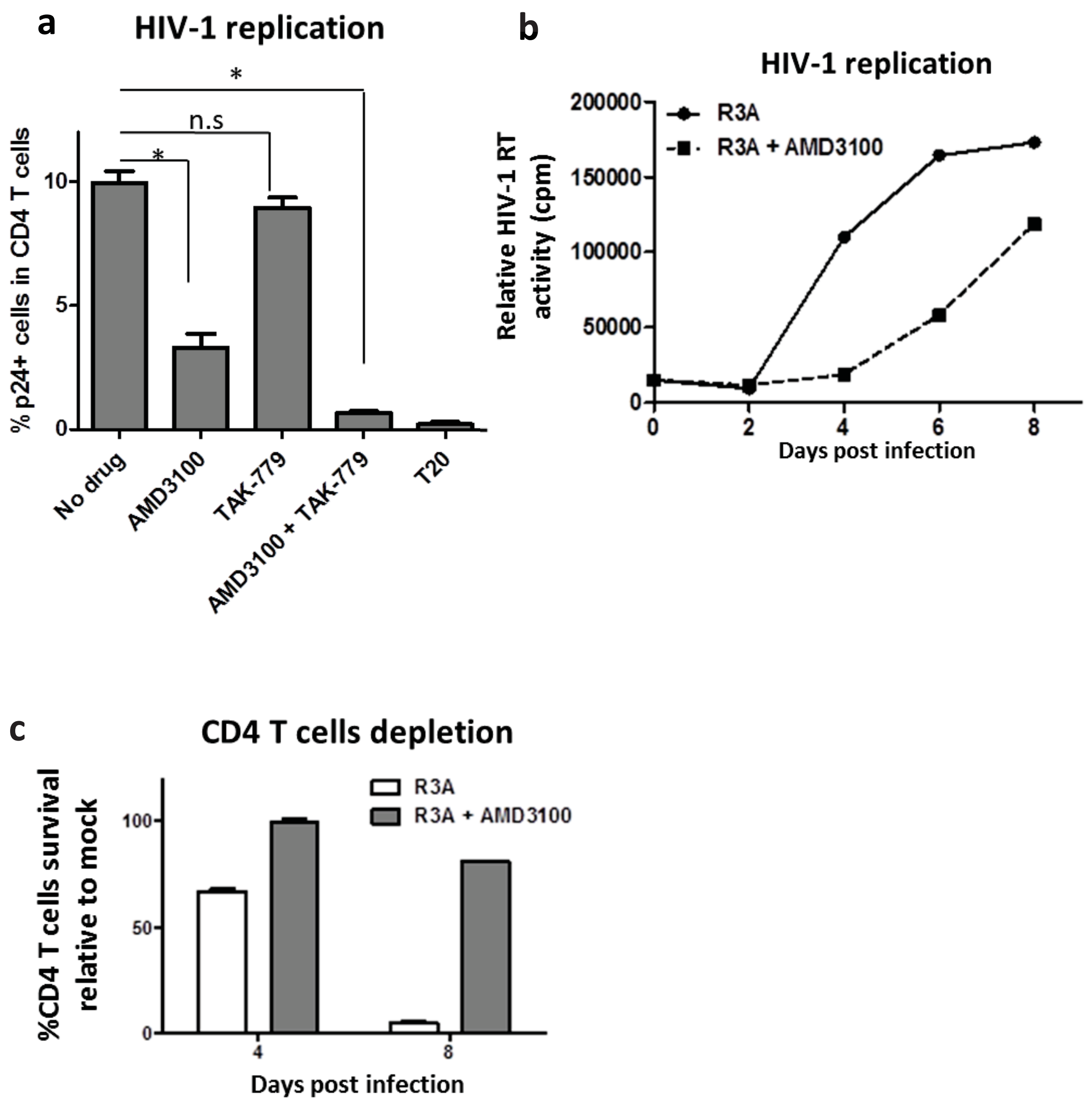


**Figure S1. CXCR4 antagonist AMD3100 inhibits R3A replication**

**a** PBMCs were treated with the indicated drugs before infection with R3A, and viral infection efficiencies were measured by %p24+ cells with FACS analysis at 4 days post infection. Both AMD3100 (2µM and TAK-779 (5µM) were used at >IC90 dose as determined before in U373-CD4-CCR5/CXCR4 cells

**b** PBMCs were treated with AMD3100 before infection with R3A and maintained after infection. HIV-1 replication was measured by extracellular HIV-1 reverse transcriptase activity in the cell supernatant.

**c** CD4 T cell depletion by R3A in the presence of AMD3100 was measured by FACS analysis as described in Figure 1b. %CD4 T cells relative to mock infected PBMCs are presented.
