# Supplementary material for: CCR5 interaction with HIV-1 Env contributes to Env-induced depletion of CD4 T cells in vitro and in vivo
Source: Retrovirology. 2016 Mar 29;13:22. doi: 10.1186/s12977-016-0255-z (PMC4812640; doi:10.1186/s12977-016-0255-z)
Supplement: Supplementary file 2 — 10.1186/s12977-016-0255-z Similar infection levels by R3A and R3A-5/6AA in PBMCs.a Infectivity of R3A and R3A-5/6AA HIV-1 strain was measured in CCR5-expressing (Top) or CXCR4-expressing (Bottom) U373 MAGI cells. Viral infectivity was presented as infectious units per ng of p24. b PBMCs were infected with 20ng p24 of R3A or R3A-5/6AA virus stock, and viral replication kinetics was analyzed by intracellular p24 staining. Representative FACS plots of p24 staining at 6dpi are presented. Supplementary to Fig. 3a. [file 12977_2016_255_MOESM2_ESM.pdf]

Figure S2

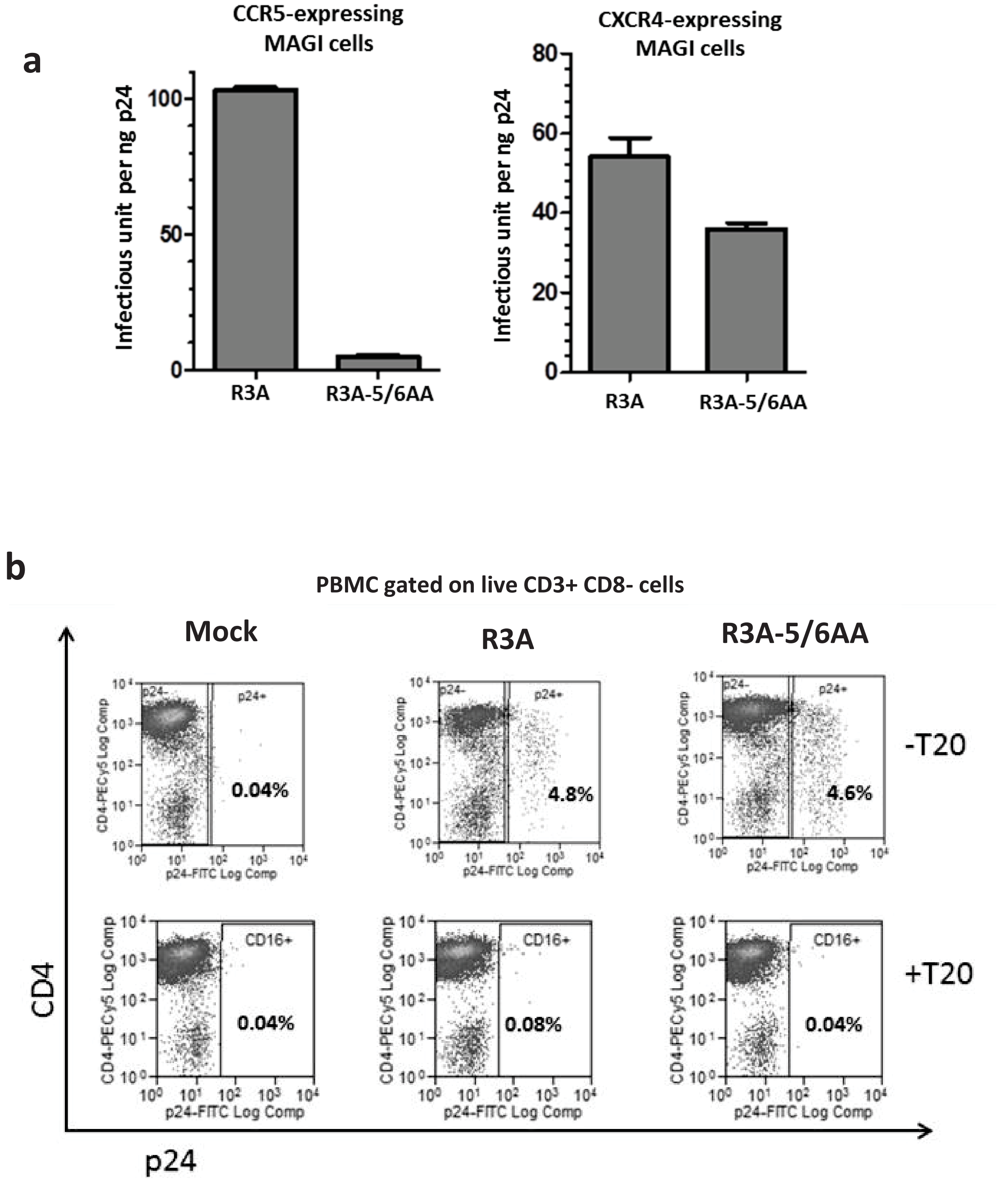

Figure S2. Similar infection levels by R3A and R3A-5/6AA in PBMCs

a Infectivity of R3A and R3A-5/6AA HIV-1 strain was measured in CCR5-expressing (*Top*) or CXCR4-expressing (*Bottom*) U373 MAGI cells. Viral infectivity was presented as infectious units per ng of p24.

b PBMCs were infected with 20ng p24 of R3A or R3A-5/6AA virus stock, and viral replication kinetics was analyzed by intracellular p24 staining. Representative FACS plots of p24 staining at 6dpi are presented. Supplementary to Fig. 3A
